# Transcriptomic Analysis of the Aged Nulliparous Mouse Ovary Suggests a Stress State That Promotes Pro-Inflammatory Lipid Signaling and Epithelial Cell Enrichment

**DOI:** 10.3390/ijms25010513

**Published:** 2023-12-30

**Authors:** Carlos Chacón, Constanza Mounieres, Sandra Ampuero, Ulises Urzúa

**Affiliations:** 1 Laboratorio de Genómica Aplicada, Departamento de Oncología Básico Clínica, Facultad de Medicina, Universidad de Chile, Santiago 8380453, Chile; chacon.biotec@gmail.com (C.C.); cmounieres@hotmail.com (C.M.); 2 Programa de Virología, Instituto de Ciencias Biomédicas, Facultad de Medicina, Universidad de Chile, Santiago 8380453, Chile; sampuero@u.uchile.cl

**Keywords:** ovarian cancer, risk, nulliparity, age, mouse model

## Abstract

Ovarian cancer (OC) incidence and mortality peaks at post-menopause while OC risk is either reduced by parity or increased by nulliparity during fertile life. The long-term effect of nulliparity on ovarian gene expression is largely unknown. In this study, we describe a bioinformatic/data-mining analysis of 112 coding genes upregulated in the aged nulliparous (NP) mouse ovary compared to the aged multiparous one as reference. Canonical gene ontology and pathway analyses indicated a pro-oxidant, xenobiotic-like state accompanied by increased metabolism of inflammatory lipid mediators. Up-regulation of typical epithelial cell markers in the aged NP ovary was consistent with synchronized overexpression of *Cldn3*, *Ezr*, *Krt7*, *Krt8* and *Krt18* during the pre-neoplastic phase of mOSE cell cultures in a former transcriptome study. In addition, 61/112 genes were upregulated in knockout mice for *Fshr* and for three other tumor suppressor genes (*Pten*, *Cdh1* and *Smad3*) known to regulate follicular homeostasis in the mammalian ovary. We conclude that the aged NP ovary displays a multifaceted stress state resulting from oxidative imbalance and pro-inflammatory lipid signaling. The enriched epithelial cell content might be linked to follicle depletion and is consistent with abundant clefts and cysts observed in aged human and mouse ovaries. It also suggests a mesenchymal-to-epithelial transition in the mOSE of the aged NP ovary. Our analysis suggests that in the long term, nulliparity worsens a variety of deleterious effects of aging and senescence thereby increasing susceptibility to cancer initiation in the ovary.

## 1. Introduction

Sporadic ovarian cancer (OC), i.e., that without any familial or hereditary component, ranks as the major cause of death by gynecological cancer worldwide. OC usually progresses with unspecific symptoms so that most cases are diagnosed at an advanced stage. In addition, methods for effective OC early detection have not been developed yet [1]. Among OC risk factors, reproductive history plays a major role. Full term pregnancies and the number of years of oral contraceptive consumption proportionally reduce OC risk. Breastfeeding has been also linked to low OC risk. In contrast, continuous ovulatory cycles during fertile life—as in nulliparity—are associated with increased OC risk [2]. The incessant ovulation hypothesis—formulated half a century ago—lies behind the effect of nulliparity on OC risk. In this condition, the occurrence of continuous ovulatory cycles implies repetitive events of local inflammation and stressful, tear and repair that might accumulate DNA damage to the ovarian surface epithelium (OSE) and to the distal fallopian epithelium, the major cell type candidates as possible origins of OC [3].

In addition to parity history, age is a major OC risk factor. In fact, since the mortality and incidence of OC markedly increases after menopause, the long-term effect of reproductive history on OC risk should be investigated in the aged ovary. The major underlying cause of reproductive aging is the depletion of the ovarian follicle reserve with subsequent disruption of the gonadal–hypothalamic–pituitary axis. This leads to decreased circulating levels of steroid hormones and increase of the pituitary gonadotropins follicle stimulating hormone (FSH) and luteinizing hormone (LH) [4].

At the local ovarian level, the stroma, the OSE, vascular endothelial cells and inclusion cysts of post-menopausal ovaries retain steroidogenic activity [5] and express receptors for estrogen (ER alpha), androgen [6] and gonadotropins (FSHR, LHR) [7]. Age-dependent follicle depletion in the ovary is closely linked to increased redox imbalance [8] and inflammation [9,10]. However, different to what is known in the post-menopausal mammalian gland regarding reproductive history and breast cancer risk [11,12], the long-term effects of pregnancy and nulliparity on gene expression of the human post-menopausal ovary are largely unknown to date.

Aimed at filling this void, a previous transcriptomic study conducted by our laboratory suggested an increased residual follicle reserve and enhanced immune surveillance in the aged, post-reproductive multiparous (MP) ovary of C57BL/6 mice [13]. As a continuation of that work, here we present a comprehensive body of literature, and bioinformatic and data-mining analysis of the counterpart gene set of higher expression levels in the aged nulliparous ovary, the condition of high OC risk. Overall, the results indicate a xenobiotic-like, pro-oxidant and inflammatory state accompanied by an enriched content of epithelial cells which is consistent with clefts and cysts commonly observed in the aged ovary [5]. This suggests a mesenchymal–epithelial transition of the OSE that might represent early steps in ovarian carcinogenesis.

## 2. Results and Discussion

Our previous work showed higher expression of genes related to residual follicular activity and enhanced macrophage/neutrophil immunosurveillance in the aged MP mouse ovary [13]. Here we show an ontology, pathway and datamining analysis of the 112 DEGs of increased transcription in the aged NP ovary, which is the high-risk condition for OC [2]. The gene expression profile of aged MP ovary was used as reference.

### 2.1. A Xenobiotic-like Stress State in the Aged NP Ovary

**Figure 1:**
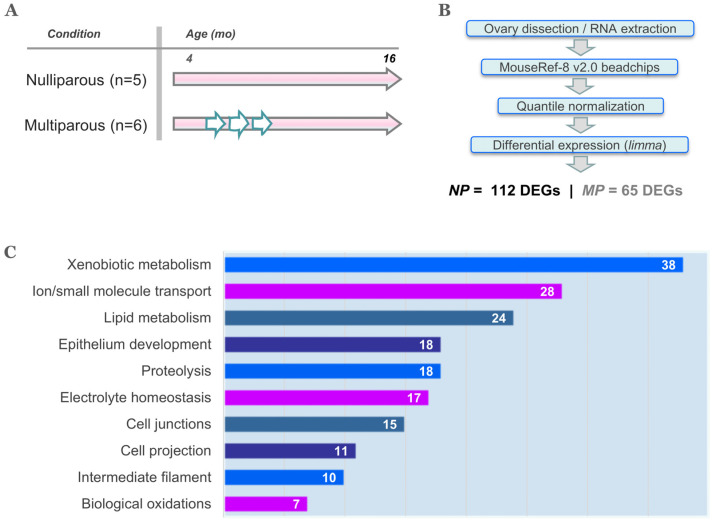
Previous study, data processing and canonical analysis of DEGs in the aged NP ovary. (**A**) Two C57BL6 female mice cohorts were maintained in nulliparous (NP) and multiparous (MP) breeding regimens from 4 through 16 months old. Short overlapped white arrows depict gestation plus lactation periods in MP mice. (**B**) Total ovarian RNA from the two conditions was analyzed with Illumina™ beadchip expression microarrays resulting in 177 differentially expressed genes (DEGs) between the NP and MP conditions [13]. (**C**) Summary of a gene set enrichment analysis (GSEA) of the 112 DEGs of higher expression in NP ovaries, using the MSigDBv7.3 database (details in Methods and Table 1). The gene number associated to each functional theme is indicated in the respective bar, and their identities described in Table 1.

GO analysis, canonical pathways and curated datasets indicated *xenobiotic metabolism* as the top biological theme with 38 genes out of 112, ~34% of the gene list (Figure 1C and Table 1). Of these, twenty-six were part of the GSEA modules # 23 (liver genes: metabolism and xenobiotics), # 24 (fetal liver genes: metabolism and xenobiotics) and # 88 (heart, liver, kidney and pancreas metabolic and xenobiotic response genes), all modules with FDR q-value< 5.9 × 10^−8^. The additional 12 genes were indexed under the GO terms “response to abiotic stimulus” and “glutathione metabolic process”. Although the formal definition of xenobiotic describes an agent of exogenous origin, here the aged NP ovary apparently develops a xenobiotic-like state promoted by endogenous substances arising as a result of age-related cellular dysfunction. One candidate substance would be lipofuscin, an amorphous, heterogeneous mixture of oxidized lipids, proteins, carbohydrates and metal ions which accumulates in various aged organs [14]. Consistently, in a previous study we detected a higher lipofuscin level in the aged NP relative to the aged MP ovary [15]. Lipofuscin is a senescence marker thought to accumulate because of age-impaired lysosomal and proteasomal function [16]. As stated above, a major determinant of organelle dysfunction in ovarian cells is the gradual decline of the oocyte/follicle reserve that leads to redox imbalance promoting oxidative damage of macromolecules as the ovary ages [8]. A further contributor to oxidative stress in the aged NP ovary, is the presence of hemosiderin aggregates [15] that might release redox active iron. Thus, it can be proposed that the aged NP ovary develops a detoxifying state that parallels or leads to senescence. Consistent with the above idea, expression of glutathione transferases *Gstm6* and *Gsta3*, glutathione peroxidase *Gpx3*, alcohol dehydrogenase *Adh7* and aldehyde dehydrogenases *Aldh1a2* and *Aldh1a7* were higher in the aged NP-compared to the MP ovary. Reduced glutathione (GSH) is a substrate of GPX and GST enzymes to detoxify H_2_O_2_ and toxic compounds while ADH and ALDH enzymes metabolize reactive aldehydes derived from lipid peroxidation [17]. If high GPX and/or GST expression correlates with elevated enzyme activities, GSH would be depleted resulting in a low GSH/GSSG ratio, which is typical of oxidative stress.

The second top theme was *ion/small molecule transport* consisting of 28 DEGs (Figure 1C and Table 1). An important subset comprised six transmembrane solute carriers including *Slc1a1*, *Slc7a5* and *Slc7a11* involved in the uptake of glutamate/aspartate, neutral bulky amino acids and cystine, respectively. In addition, *Slc5a4* codes for a sodium–glucose co-transporter, *Slc2a3* for the facilitative glucose transporter Glut3 and *Slc13a4*, for a sodium–sulfate co-transporter. Interestingly, *Slc1a1* is a versatile solute carrier which can also transport cystine, so it is linked to glutathione homeostasis [18] like *Slc7a11*. These findings suggest that the aged NP ovary might increase glutathione synthesis to cope with endogenous oxidative stress.

Further transport related genes included *Camk2b*, *Fxyd4* and *Atp12a* which participate in cation transport by P-type ATPases while *Camk2b*, *Adora1*, *Avpr1a* and *Gng13* are related to G-protein coupled receptor signaling. *Atp12a* codes for a non-gastric H^+^/K^+^-ATPase [19] whereas *Avpr1a* codes for the vascular vasopressin receptor 1A, both proteins involved in homeostasis of water and solutes, a process known to be impaired with age [20]. As shown here in the aged NP ovary, *Avpr1a* was found upregulated in the kidneys of aged Fischer 344 rats [21]. Relatedly, the aged NP ovary also overexpressed the transmembrane water channels aquaporin paralogs *Aqp1* and *Aqp5*, both related to the *Gng13* (G-gamma 13) gene under the Reactome term “aquaporin-mediated transport” (R-HSA-445717). In addition to water, aquaporins also transport ions, gasses, small molecules, and reactive species including H_2_O_2_ and NO [22]. In the fertile-age ovary, *Aqp1* was localized in the endothelial cells of capillaries whereas *Aqp5* was found in the primordial follicles and in granulosa cells of developing follicles [23]. Aquaporins have been implicated in OC progression and chemoresistance by various mechanisms including oxidative stress [24].

**Figure 2:**
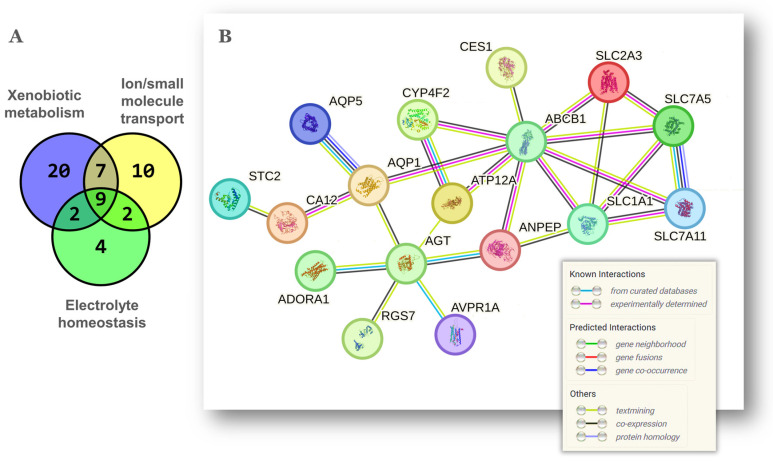
Overlap and gene network among xenobiotic metabolism, ion/small molecule transport and electrolyte homeostasis. (**A**) Venn diagram to determine the gene coincidence between 3 of the functional themes of Figure 1C. The gene lists of each theme are described in Table 1. (**B**) Known and predicted relationships among the 20 coincident genes shown as a gene network obtained with STRING v12.0 (0.35 confidence score; unconnected nodes removed). The meaning of color lines depicting gene interactions is shown at the bottom-right and was adapted from the software’s output.

Based on the above-described function of genes *Slc5a4*, *Slc13a4*, *Avpr1a*, *Aqp1* and *Aqp5*, the theme *electrolyte homeostasis* (Figure 1C and Table 1) seemed obviously related to *xenobiotic metabolism* and *transport of ion/small molecules*. The gene overlap between these three themes is shown in the Venn diagram of Figure 2A. Nine genes were common to the three themes while 20 genes were common to at least two. The protein–protein interaction network shown in Figure 2B was built with these 20 genes. Interestingly, the solute carrier proteins coded by *Slc1a1*, *Slc7a5*, *Slc7a11* and *Slc2a3*, formed a major hub around *Abcb1*, which is the gene for the P glycoprotein/MDR1, an ATP-dependent efflux pump of xenobiotic molecules with a protective role against chemotoxicity in the ovary [25] and associated with multidrug resistance in several cancers including OC [26]. Additional *Abcb1* connectors were the *Atp12a* H^+^/K^+^-ATPase and the *Aqp1* aquaporin. Both *Abcb1* and *Aqp1* have been found coexpressed in diverse cell toxicity models [27,28]. In turn, *Aqp1* was linked to the angiotensinogen *Agt*, which codes for the angiotensin-II (Ang-II) precursor. A further member of the renin–angiotensin system (RAS) in the network of Figure 2B was the aminopeptidase *Anpep,* which converts the angiotensin Ang-III to Ang-IV. Moreover, an alternative KEGG analysis of the original list of 112 mouse genes indicated a significant enrichment of the mouse RAS including the genes *Klk1*, *Mcpt4*, *Anpep*, *Agt* and *Klk1b5*. The local ovarian RAS plays important roles in follicular development, oocyte maturation, steroidogenesis, luteogenesis and luteolysis during fertile age [29] but becomes chronically activated with aging leading to ROS production though increased Ang-II activity [30]. Chronic behavioral stress is associated with high ovarian and serum Agt and Ang-II levels resulting in granulosa cell apoptosis and decreased ovarian reserve [31]. This latter feature is consistent with our previous report showing that the aged NP ovary displays a smaller residual ovarian reserve relative to the aged MP ovary [13]. Finally, *Aqp1* was also linked to *Car12*, the carbonic anhydrase XII, an enzyme that along with *Car9* catalyzes the reversible hydration of CO_2_ to bicarbonate and protons thus lowering cellular pH [32]. Both enzymes are upregulated by hypoxia, another feature of cellular aging [33] recently linked to depletion of the ovarian reserve [34], hence again consistent with our previous report [13]. In summary, the latter group of interconnected proteins suggest a stress-state characterized by altered body fluid, electrolyte homeostasis and acid–base balance in the NP ovary.

### 2.2. Genes Associated to Metabolism of Pro-Inflammatory Lipid Mediators

**Figure 3:**
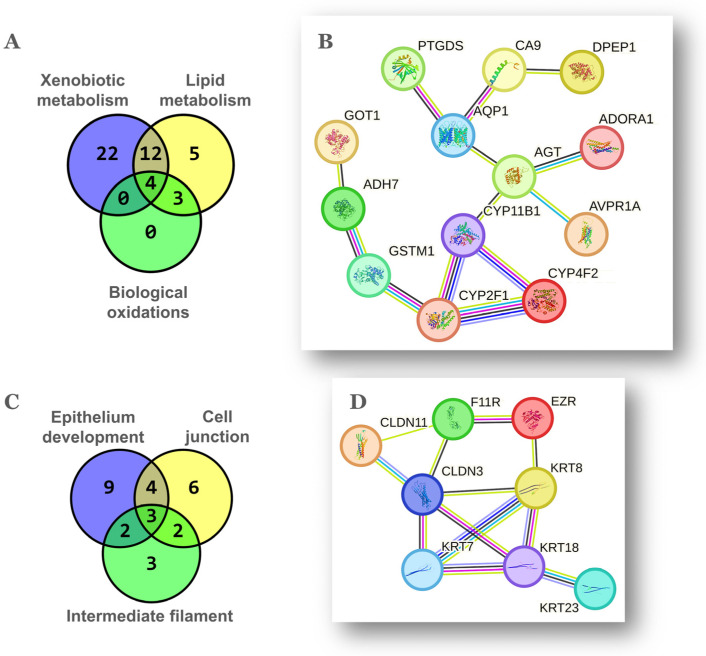
Overlap and gene networks among oxidative metabolism and epithelium related themes. Venn diagrams showing the gene coincidence between themes related to oxidative metabolism (**A**) and to epithelium (**C**). Detailed gene groups are shown in Table 1. Gene networks among the 20 coincident genes of themes related to oxidative metabolism (**B**) and among the 11 coincident genes of themes related to epithelium (**D**). Parameters of STRING v12.0 analysis and meaning of color-coded line interactions as in Figure 2B.

The third ranking theme upregulated in the aged NP ovary was *lipid metabolism* with 24 genes (Figure 1C, Table 1), 16 of which overlapped with *xenobiotic metabolism* (Figure 3A). In turn, all 7 genes classified under *biological oxidations* were part of *lipid metabolism* (Figure 3A). As shown in Figure 3B, the p450 cytochromes *Cyp11b1*, *Cyp2f2* and *Cyp4f15* (human orthologs *CYP11B1*, *CYP2F1* and *CYP4F2*) were extensively interconnected according to their cognate functions. *Cyp11b1* codes for the mitochondrial steroid 11-beta hydroxylase involved in ovarian synthesis of glucocorticoids, suggestive of a hormonal stress response [35]. The p450 cytochrome *Cyp2f2* (human *CYP2F1*) detoxifies endogenous agents and xenobiotics. *Cyp2f2* was connected to *Gstm6* (human *GSTM1*), the latter catalyzes the conjugation of prostaglandins A2 and J2 with glutathione [36]. In turn, *Gstm6* was connected to the alcohol dehydrogenase *Adh7*, an enzyme that oxidizes retinol to retinoic acid and is induced by FSH in the mouse ovary [37]. Retinoids are dietary lipids important for ovarian function such as follicle homeostasis and steroidogenesis [38]. In addition to *Adh7*, the genes *Aldh1a2* and *Ptgds* are classified under the function “retinol binding” in GO. 

The cytochrome *Cyp4f15* (human *CYP4F2*) along with the prostaglandin-D2 (PGD2) synthase *Ptgds* and the dipeptidase *Dpep1* are involved in the metabolism of arachidonic acid (AA), the unsaturated fatty acid precursor of proinflammatory lipid mediators such as prostaglandins, thromboxanes, and leukotrienes. *Cyp4f15* hydroxylates AA to form 20-hydroxyeicosatetraenoic acid (20-HETE), a vasoactive, pro-hypertensive agent whose action is enhanced by androgens and linked to the RAS [39]. As shown above, ovarian RAS genes including *Agt* were also upregulated in the aged NP ovary (Section 2.1). On the other hand, expression of *Ptgds* inhibits granulosa cell proliferation in the adult ovary [40] while PGD2 synthesis increases in the rat ovary in response to the toxicant triptolide [41]. Similarly, the dipeptidase *Dpep1* enzyme converts leukotriene-D4 to -E4 and also acts as a neutrophil adhesion molecule promoting inflammation [42].

Another subset of genes (*Agt*, *Avpr1a*, *Pik3r3* and *Sphk1*) were involved in the phospholipase D signaling pathway. The latter two did not appear connected to the network of Figure 3B, but may play important roles in the aged NP ovary. *Pik3r3* codes for the regulatory subunit of the phosphatidylinositol-3-kinase, which coupled with PDK-1/Akt, PDK-1/PKC or mTOR/Akt can prevent granulosa cell apoptosis during follicle atresia [43]. *Sphk1* codes for an enzyme catalyzing the synthesis of sphingosine-1-phosphate, a compound with antiapoptotic capacity on primordial ovarian follicles [44]. However, in the context of OC, high *Sphk1* expression is associated with growth, chemoresistance and metastasis [45].

Compared to the aged MP ovary [13], steroid metabolism was less relevant among genes overexpressed by the aged NP ovary; just a couple of exceptions were the lathosterol desaturase *Sc5d*, member of the cholesterol synthesis pathway, and *Stard5*, an ER-stress gene involved in regulation of membrane and intracellular cholesterol homeostasis [46].

### 2.3. A Possible Mesenchymal-to-Epithelial Transition Signature in the Aged NP Ovary

Noteworthy, despite we analyzed the transcriptomes of whole ovaries, 29/112, ~26% of DEGs upregulated in the aged NP condition were enriched in the themes *epithelium development*, *cell junction* and *intermediate filament* (Table 1). This finding suggests that the aged NP ovary has a significant content of epithelial cells. The coincident genes between the three above-mentioned themes were *Agt*, *Cldn3*, *Cldn11*, *Ezr*, *F11r*, *Krt7, Krt8*, *Krt18*, *Krt23*, *Nefh* and *Slc7a11* (Figure 3C), eight of which become connected in the network of Figure 3D. In this regard, the post-menopausal ovarian cortex accumulates clefts and inclusion cysts, structures of epithelial morphology derived from OSE invaginations towards the stroma and thought to be precursor lesions for certain OC types [47]. These cysts correlate positively with age and ovulation count in mice [48]. Moreover, while the OSE displays both mesothelial and epithelial characteristics, the ovarian inclusion cysts predominantly express epithelial markers, suggesting a mesenchymal–epithelial transition [49].

The cytoskeletal keratins and additional proteins that form intermediate filaments in single-layer epithelia, provide resistance not only against mechanical but also various types of stress such as those occurring with inflammation and aging [50,51]. In addition, the claudins *Cldn3*, *Cldn11*, *Cldn15* plus the *F11r* receptor are components of the intercellular tight junctions that physically connect epithelial cells while the *Ezr* protein links the cytoskeleton to the apical plasma membrane. *Ezr* and *Krt8* are expressed by the normal mouse OSE (mOSE) irrespective of physiological state [52]. The expression of *Krt8* was assayed and quantified by IHC in ovarian sections (Figure S1) and levels agreed with the FC detected between NP and MP ovaries by the array method. Moreover, in a previous transcriptomic study conducted in our laboratory, *Krt8*, *Krt18*, *Cldn3*, *Ezr* and *F11r* were upregulated in critical passages during spontaneous transformation of mOSE cells in culture [53]. Importantly, *Ezr* is expressed by cultured human OSE cells and ovarian cleft cells of post-menopausal human ovaries [54].

**Figure 4:**
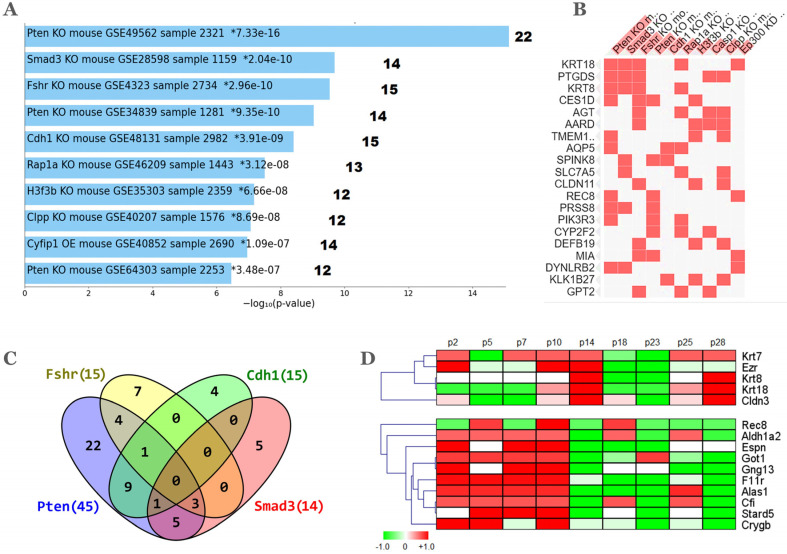
GEO datamining of KO mouse models. The 112 DEGs were mined in the Enrichr database *crowd* tab, *gene perturbations from GEO up* output. (**A**) The top 10 mouse KO datasets ranked by *adj-p* values. KO gene symbol and GEO accession is indicated within each bar. The number at the right indicates the gene count from the 112 DEGs which are contained in each dataset. (**B**) Overlap of the top 20 genes in the 10 KO datasets shown in (**A**). (**C**) Gene coincidence between the top 4 KOs; (**D**) Selected heatmaps of 46/112 genes that were found in the transcriptome dataset of pre-malignant, cultured mOSE cells. Labels p2 to p28 indicate passage 2 through passage 28 log_2_ fold change averages of 4 replicate two-channel arrays [53].

Upregulation of claudins *Cldn3* and *Cldn11* was also observed in the ovaries of knockout m for the FSH receptor (FORKO), a model that develops serous papillary adenocarcinoma with age [55]. If we flip our previous data on the aged MP ovary [13], we see that *Fshr* is downregulated in the NP relative to the MP ovary, a situation that mimics the FORKO ovary [55]. Hence, an inverse relationship seems to take place: *Fshr* downregulation is associated with overexpression of claudins *Cldn3*, *Cldn11* and *Cldn15* in the NP ovary while the opposite occurs in the MP ovary [13]. Additional upregulated genes in the FORKO ovary were coincident with part of the 112 gene set of aged NP ovaries (see Section 2.5, Figure 4).

Further significant themes were *proteolysis* and *cell projection* with 18 and 11 genes, respectively. In the *proteolysis*-related genes, all except *Eppin* and *Fbxl22* are glycoproteins and all except *Edem2* and *Fbxl22* have extracellular locations. In addition, 7 genes code for peptidases, 6 for peptidase inhibitors and 3 were also part of the RAS. Protease activity is a feature of the so-called SASP, the senescence-associated secretory phenotype [56].

### 2.4. Gene Expression Datamining of the Ovarian NP Signature

Mouse gene knockouts (KO) provide the opportunity to address the impact that gene inactivation/deletion might have on a certain phenotype and its cognate transcriptomic profile. With this aim, the enrichment of the 112 DEGs upregulated in the aged NP ovary was sought in KO signatures curated from the GEO database. A meaningful analysis should be restricted to only up-regulated genes as a result of the KO. A total of 99 GEO signatures were found and ranked according to *adj p* < 0.05. Figure 4A shows the top 10 ranked signatures and their respective gene count in the 112 DEGs while Figure 4B shows a partial list of coincident genes. Interestingly, among the top-4 KOs, all except *Fshr*, are well-known tumor suppressor genes implicated in the homeostasis of ovarian follicles (see Section 2.5 below). The Venn diagram of Figure 4C shows a total of 23 coincident genes with at least one coincidence between any pair of the top 4 KOs. The largest overlap was 9 genes between the *Pten* and *Cdh1* KOs while the genes *Krt8*, *Krt18* and *Ptgds* were common among 3 (*Pten*, *Fshr* and *Smad3*) of the 4 KOs. No gene was common to the 4 KOs.

The phosphatase and tensin homolog deleted on chromosome 10 (*Pten*) gene KO datasets GSE49562, GSE34839 and GSE64303 comprised 22, 14 and 12 genes, respectively. Two additional *Pten* KOs not shown (datasets GSE64303 and GSE47520) were ranked in 31st and 33rd places and provided 10 and 9 additional genes, respectively. There was some gene overlap among these five *Pten* datasets, so that their combination resulted in 45/112 genes representing ~40% of the DEGs upregulated in the NP condition. *Pten* is a negative regulator of the phosphatidylinositol 3-kinase (PI3K) pathway and its decreased expression in the oocyte induces massive follicle activation thus depleting the ovarian reserve [57]. Inactivation of *Pten* plus *Tp53* and *Rb1* in the mOSE induced epithelial hyperplasia and micropapillary carcinoma, while further *Cdh1* inactivation enhanced tumor dissemination and metastasis [58].

The second-ranked KO was *Smad3*, a transcription factor that forms a complex with other Smad proteins to bind DNA. The Smad family of transducer proteins are involved in TGF-beta signaling, a pathway relevant to follicular homeostasis [59]. Importantly, *Smad3* has been found to inhibit mOSE proliferation [60]. The third-ranked KO was *Fshr* and corresponds to the FORKO model mentioned above. These two enriched KO signatures in genes overexpressed by the aged NP ovary are consistent with our previous report showing up-regulation of *Fshr* and several genes of the TGF-beta family in the aged MP ovary [13]. The fifth KO was *Cdh1* which codes for E-cadherin, a multi-domain cell-cell adhesion protein widely used as marker of epithelial cells that has been involved in ovarian follicle integrity [61]. The detected *Cdh1* KO signature (GSE48131) is derived from a double conditionally deleted *Cdh1*-*Tp53* model of endometrial tumors [62] that might not accurately represent spontaneous pre-neoplastic changes of the OSE. Indeed, increased *Cdh1* expression is observed in just one quarter of inclusion cysts of mixed (columnar/ciliated & cuboidal/flat) phenotype in the human OSE [49] whereas spheroids formed with normal OSE cells express low *Cdh1* levels as compared with those formed with OC cells [63].

Among these top 4 KOs, the FORKO i.e., *Fshr* corresponded to the only dysregulated gene between the aged NP and the aged MP ovary [13]. Canonical *Fshr* signaling occurs through the Gs/cAMP/protein kinase A pathway, but several other pathways might be intertwined with the canonical one in the mammalian gonads [64]. A relevant link between *Fshr* and *Pten* is suggested by increased expression and activity of *Pten* in response to FSH stimulation, thus restricting proliferation of prepubertal Sertoli cells [65]. In addition, oncogenic Akt signaling is inhibited by nuclearly localized *Cdh1* which stabilizes *Pten* by preventing its ubiquitination [66] while TGF-*β* induces nuclear translocation of *Smad3* which then participates in *Pten* gene transcription thereby inhibiting PI3K/AKT signaling [67].

### 2.5. Follicle Depletion and the Pre-Neoplastic mOSE

The inverse relationship observed between upregulation of claudins and downregulation of *Fshr* in the aged NP ovary—which resembles the FORKO model—also extends to alpha-inhibin and *Krt8*, markers of granulosa and epithelial cells respectively, in the related germ-cell-deficient *Wv* mouse model [68]. Due to a *c-kit* point mutation, ovaries of Wv/Wv females contain less than 1% of the normal oocyte count at birth so that ovarian follicles are rapidly depleted by reproductive age with concomitant histological changes such as epithelial invaginations, inclusion cysts, papillomatosis, and benign ovarian tumors [69]. These authors suggested that “granulosa cells may be part of a negative paracrine signal to suppress ovarian epithelial proliferation”. Therefore, increased expression of epithelial genes in the NP ovary might be the consequence of a lower follicular content, further than decreased by age only, as we previously reported [13]. With this idea, the 112 gene list was queried in a transcriptome dataset tracking the expression changes that mOSE cells undergo in culture [53]. Figure 4D shows heatmaps of 15 out of the 46 DEGs of the aged NP ovary that were present in the dataset of mOSE cultures from passage 2 through passage 28. Notably, the epithelial genes *Cldn3*, *Ezr*, *Krt7*, *Krt8* and *Krt18* in the upper heatmap were coordinately up-regulated at passage 14, a stage where proliferative stress and cytokinetic defects leads to in vitro mOSE tetraploidy that precedes development of a malignant phenotype [53,70]. A total of nine other genes of the lower heatmap were upregulated from early 2-to-10 passages and then switched off in advanced passages. These included the OSE stem cell marker *Aldh1a2*, the receptor *F11r* that regulates tight junction assembly in epithelia, and the actin-bundling protein *Espn*, that interacts with membrane–cytoskeleton linkers such as *Ezr* to participate in microvilli biogenesis [71].

### 2.6. The Status of Selected Genes Expressed by the Aged NP Mouse Ovary in Human OC

**Figure 5:**
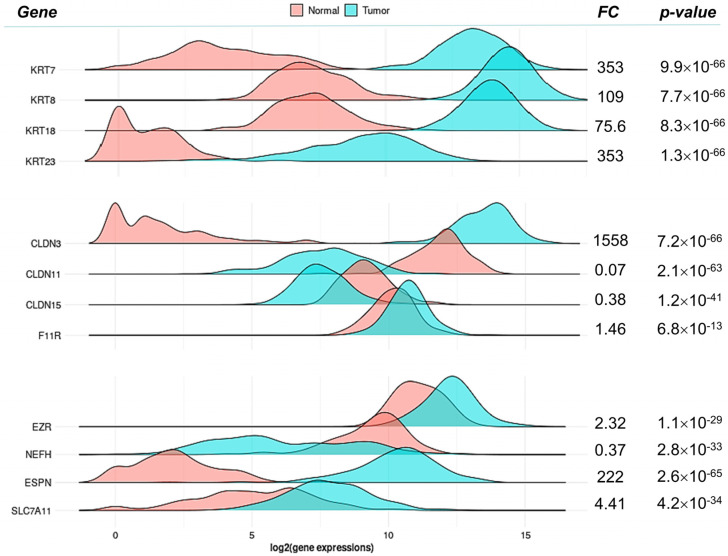
Selected gene expression in human ovarian carcinomas. A subset of 12 DEGs were queried in TNMplot as their respective human orthologs (see Methods). Gene expression density plots are shown for the indicated genes (left) in 133 normal and 374 human ovarian cystadenocarcinoma samples (“Tumor” label). The fold change (FC) corresponds to the direct ratio between mean expression values for each group; *p* values were obtained from a Mann–Whitney U test. Density plots are supported by boxplots of Figure S2.

While we remark that the aged NP ovary develops an apparent cellular stress state that not necessarily implies cancer initiation, we were interested to gain insight about the status in human OC tumors of the corresponding orthologs of a few genes overexpressed in the aged mouse NP ovary. A total of twelve genes coding for structural proteins of the intermediate filaments and cell junctions were selected for this analysis. The FC values (tumor/normal) ranged from 1158 (*CLDN3*) to 0.07 (*CLDN11*). Importantly, 9/12 genes were upregulated in tumors, i.e. same as found in the aged NP ovary (Figure 5). Notably, all the *KRT7*, *KRT8, KRT18* and *KRT23* cytokeratins were highly overexpressed in tumor versus normal samples (mean FCs 353, 109 and 75.6 and 353, respectively), an observation consistent with their involvement in epithelial mesenchymal transition by OC cells [72,73]. The claudin *CLDN3* fit the same pattern as keratins in contrast with the claudins *CLDN11* and *CLDN15* in which the healthy ovarian tissue showed higher levels than tumors. Like the keratins, *CLDN3* overexpression was found to increase OC invasion and survival linked to *MMP2* expression [74]. The opposite pattern of *CLDN11* is coherent with a proposed role as tumor suppressor in gastric and nasopharyngeal carcinoma, where it was downregulated by hypermethylation [75,76]. The similar low-expression pattern of *CLDN15* in tumors might be linked to reduced overall survival in OC [77]. Among the remaining genes, *NEFH*, encoding the neurofilament heavy polypeptide, resembles the *CLDN11* and *CDLN15* pattern whereas *F11R* (alias JAM1), *EZR* (alias Villin), *ESPN* and *SCL7A11* resemble the keratins and *CLDN3* pattern. In summary, a significant fraction of genes up-regulated in the aged NP ovary are also upregulated in OC. Interestingly, as shown in Figure 4D, the genes *KRT7*, *KRT8*, *KRT18* and *CLDN3* are up-regulated in mOSE cells in a biphasic mode: first at passage 14, which is a preneoplastic stage and later at passage 28, which is a malignant phase. The latter might reflect their upregulation in human OC. In contrast, *EZR* overexpresses only at passage 14 but does not “re-appear” at late passage 28, which is consistent with its pattern in Figure 5. We emphasize that the biological distance between the aged NP ovary and the neoplastic ovary does not support a straight comparison. The occurrence of mutational events and evasion of cell senescence in the NP ovary would be requisites for further transformation and carcinogenesis.

## 3. Materials and Methods

### 3.1. Experimental Design, Data Collection and Differential Expression

This study was approved by the Bioethics Committee, Faculty of Medicine, University of Chile (Protocol #0536). As shown in Figure 1A, two cohorts of C57BL/6 female mice were housed in multiparous (MP) and nulliparous (NP) conditions as previously described [13,15,78]. Mice were fed a standard rodent diet plus filtered water ad libitum all over the course of the experiment. General healthcare by a specialized DMV was provided regularly. The reproductive performance of the MP group was reported elsewhere [78]. By ~16 months old, a subset of animals of each condition were euthanized, ovaries dissected, and total RNA isolated with the All-Prep kit (Qiagen, GmbH, Hilden, Germany). After digestion with Turbo-DNAse (Ambion, Foster City, CA, USA), RNA was precipitated with NaAc/ethanol and stored at −80 °C. RNA was dissolved in nuclease-free water and A260/A280nm read to determine RNA yield and purity. RNA integrity (RIN) was done with the Agilent 2100 Bioanalyzer (Agilent Tech, Santa Clara, CA, USA). All RNA samples meet RIN values > 7.5. RNA profiling was performed by Macrogen Inc. (Seoul, Republic of Korea). Briefly, total RNA (0.5 μg) was reverse-transcribed with T7 oligo(dT) primers and a second-strand cDNA obtained. T7 RNA polymerase and biotin-labeled NTPs were added to produce a biotinylated cRNA, which was hybridized to Mouse Ref-8 v.2 beadarrays for 16–18 h at 58 °C. After the addition of fluorolink streptavidin-Cy3 (Amersham, GE Healthcare, Little Chalfont, UK), fluorescent signals were detected with an Illumina confocal scanner and processed with the Illumina GenomeStudio software 2011.1. The Mouse Ref-8 v.2 (Illumina Inc., San Diego, CA, USA) is an oligonucleotide-based bead-array expression platform composed of 25,697 distinct probes covering 17,640 unique mouse coding genes. The complete annotation data for this microarray is found as Supplementary File S2. Raw microarray data were uploaded to the NCI microarray database (http://nciarray.nci.nih.gov, accessed on 31 July 2019), and there normalized by quantiles and subjected to statistical analysis. A *limma* test with multiple test adjustment (FDR, Benjamini–Hochberg method) was performed (Figure 1A) using *adj p* < 0.10 and log_2_ FC > ±0.8 as cut-offs. This resulted in 177 differentially expressed genes (DEGs) between the NP and the MP conditions [13]. Of these, 112 DEGs showed higher transcript levels in NP ovaries and are analyzed in this report (Figure 1B). The complete list of DEGs is freely available in the Supplementary Material of Urzua et al. [13].

### 3.2. Mouse–Human Orthology

The mouse–human orthology was verified through the MGI’s vertebrate homology portal (https://www.informatics.jax.org/homology.shtml, accessed on 28 August 2023) and the DIOPT ortholog finder v9 (https://www.flyrnai.org/cgi-bin/DRSC_orthologs.pl, accessed on 30 August 2023). With the latter tool, ortholog weighted scores were high (range 20.3–10.9) for 101/112 of genes, moderate (range 12.8–4.9) for 10/112 and low (0.9) for 1/112. This low score gene (*5031410I06Rik*) was excluded in further analyses. In addition, no human ortholog was found for *Svs5*, coding for seminal vesicle secretory protein 5. The three mouse *Klk1* paralogs *Klk1b4*, *Klk1b5* and *Klk1b27* were converted to the unique human ortholog *KLK1* gene (see Supplementary File S1).

### 3.3. Canonical Enrichment

Analyses of pathways, gene ontology (GO) and curated thematic gene set were conducted in the Gene Set Enrichment Analysis (GSEA), Molecular Signatures Database v7.3 (http://software.broadinstitute.org/gsea/msigdb/, accessed on 31 August 2023). GSEA was queried with the 112 mouse genes as a list of gene symbols, which were automatically converted to human genes by using the option “Mouse (to be orthology-mapped)”. In addition to *Svs5* and *5031410I06Rik*, three additional genes (*Gsta3*, *Mcpt4* and *Spmip6*) were not converted. Thus, the GSEA analysis shown here was done on 103 human orthologs derived from 107 out of the initial 112 mouse genes. Gene sets selected were positional (C1), curated (C2), regulatory (C3), computational (C4), ontology (C5), immunologic signature (C6) and hallmark (H). The complete GSEA overlap results are contained in the Supplementary File S3. Selected terms compiled in Table 1 under distinct biological *themes* (leftmost header) were derived mostly from the curated (C2), computational (C4) and GO (C5) gene sets. C4 gene modules referred to as “Genes in the cancer module XX” were not considered.

### 3.4. Mining of GEO Gene Expression Signatures

The original list of 112 genes was analyzed with the knowledge discovery tool Enrich (https://amp.pharm.mssm.edu/Enrichr/, accessed on 26 July 2023), specifically the “crowd” gene set library which is derived from a crowdsourcing project aimed to identify and annotate diverse signatures of Gene Expression Omnibus (GEO; https://www.ncbi.nlm.nih.gov/geo/, accessed on 26 July 2023) database. The top four *p*-value ranked knockout records of the subset “Gene Perturbations from GEO up” were selected and their overlapping genes identified through a Boolean comparison (https://bioinfogp.cnb.csic.es/tools/venny/, accessed on 15 September 2023). Finally, the 112 gene set was manually queried in the GEO’s GSE81729 dataset, which describes transcriptomic changes of mouse ovarian surface epithelial (mOSE) cells over the course of spontaneous transformation in culture [53].

### 3.5. Gene Expression Analysis of Ovarian Carcinomas

The expression of a subset of upregulated genes in the NP ovary was explored in an expression database of human OC by means of the tool TNMplot (https://tnmplot.com/analysis/, accessed on 18 December 2023). Using the RNAseq data collection, normal samples (*n* = 133) were selected from non-cancerous patients and the tumor samples were ovarian serous cystadenoma (*n* = 374). Significance was evaluated through the Mann–Whitney U test.

## 4. Conclusions

We previously showed higher expression of genes related to residual follicular function and enhanced innate immunity in the aged MP ovary, which represents the low-risk OC condition [13]. Knowledge about nulliparity, the opposite high-risk condition, on ovarian gene expression is largely scarce to date. Here, we show that the aged NP ovary displays a multifaceted stress state characterized by elevated expression of xenobiotic-like and transport genes to maintain the antioxidant, electrolyte and acid–base homeostasis. Moreover, through up-regulation of various oxidative enzymes, this stress milieu expands to genes implicated in the metabolism of pro/anti-inflammatory lipid mediators such as arachidonic acid derivatives, retinoids and phospho- and sphingolipids. A further consequence of this stress state is the elevated expression of genes coding for structural components of epithelial cells such as intermediate filaments, cell junctions and adaptor proteins, a fact consistent with increased clefts and cysts observed in aged human and mouse ovaries that suggests a mesenchymal–epithelial transition in the mOSE as proposed in the human OSE [49]. This enriched content of epithelial cells might also reflect a depleted ovarian reserve since 3/4 of top KO models correspond to genes that contribute to the oocyte/follicle homeostasis. While many of these stress-related features of the aged NP ovary can be considered typical of age-impaired tissue homeostasis, we remark that the aged MP ovary is not affected by this stress phenotype. It could be inferred that pregnancy in the long term attenuates or delays the deleterious effects of aging and senescence thereby minimizing susceptibility to cancer initiation in the ovary.

## Figures and Tables

**Table 1:** Canonical analysis of genes overexpressed in the aged NP mouse ovary.

Theme ^a^ (# Genes)	Specific Terms ^b^	Database ^c^	Genes ^c^
*Xenobiotic metabolism* (38)	Modules 23, 24 & 88 Response to abiotic stimulus Glutathione metabolic process	CGS (C4) GO (C5) GO (C5)	*ABCB1, ADORA1, AGT, AHSG, ALAS1, ANPEP, AQP1, AQP5, AVPR1A, CA12, CA9, CES1, CFI, CHRNB1, CLDN3, COX6A2, CYP2F1, DPEP1, F11R, GADD45B, GOT1, GSTM1, KRT18, KRT7, KRT8, MAPKAPK3, PDZK1IP1, PRSS8, PTGDS, RGS7, SCX, SLC1A1, SLC2A3, SLC7A11, SLC7A5, STARD5, STC2, WWC1*
*Ion/small molecule transport* (28)	Monoatomic ion transport Transport of small molecules Transporter activity	GO (C5) CP (C2) GO (C5)	*ABCB1, ADORA1, AGT, AKR1C4, AQP1, AQP5, ATP12A, AVPR1A, CAMK2B, CES1, CHRNB1, CLDN15, CYP4F2, EPPIN, FXYD4, GNG13, PRSS8, RGS7, SLC13A4, SLC1A1, SLC2A3, SLC5A4, SLC7A11, SLC7A5, STARD5, STC2, TMEM184A, UNC79*
*Lipid metabolism* (24)	Lipid metabolic process Response to lipids Metabolism of lipids	GO (C5) GO (C5) CP (C2)	*ADH7, ADORA1, AGT, AKR1C4, ALAS1, ALDH1A2, AQP1, AVPR1A, CA9, CES1, CYP11B1, CYP2F1, CYP4F2, DPEP1, GOT1, GSTM1, MAPKAPK3, PIK3R3, PTGDS, SC5D, SLC7A5, SPHK1, STARD5, STC2*
*Epithelium development* (18)	Epithelium development Formation of the cornified envelope	GO (C5) CP (C2)	*AGT, ALDH1A2, AQP1, CA9, CES1, CLDN3, CRYGB, EZR, F11R, KRT18, KRT23, KRT7, KRT8, PRSS8, SCX, SLC7A11, UPK1A, ZIC3*
*Proteolysis* (18)	Proteolysis	GO (C5)	*ACAN, ADAMTS4, AHSG, ANPEP, AQP1, CELA3B, CFI, CLDN3, CMA1, CST9L, DPEP1, EDEM2, EPPIN, FBXL22, KLK1, PRSS8, SLC1A1, SPINK8*
*Electrolyte homeostasis* (17)	Metabolism of angiotensinogen Monoatomic ion homeostasis Circulatory system process	GO (C5) GO (C5) GO (C5)	*ABCB1, ADORA1, AGT, ANPEP, ATP12A, AVPR1A, CA12, CALB2, CES1, CMA1, CYP11B1, CYP4F2, KLK1, SLC1A1, SLC2A3, SLC7A5, STC2*
*Cell junction* (15)	Cell-cell junction Cell junction organization Tight junction interactions	GO (C5) GO (C5) CP (C2)	*AGT, CALB2, CAMK2B, CDHR3, CHRNB1, CLDN11, CLDN15, CLDN3, EZR, F11R, KRT18, KRT8, NEFH, SLC1A1, SLC7A11*
*Cell projection* (11)	Cell projection membrane Microvillus Actin based cell projection	GO (C5) GO (C5) GO (C5)	*ADORA1, AQP1, AQP5, ARHGEF4, CA9, DPEP1, ESPN, EZR, SLC7A11, SLC7A5, WWC1*
*Intermediate filament* (10)	Intermediate filament Polymeric cytoskeletal fiber	GO (C5) GO (C5)	*CFAP161, CLDN11, DYNLRB2, ESPN, EZR, KRT18, KRT23, KRT7, KRT8, NEFH*
*Biological oxidations* (7)	Biological oxidations	CP (C2)	*ADH7, CES1, CYP11B1, CYP2F1, CYP4F2, DPEP1, GSTM1*

^a^ Themes in italics were coined as keywords derived from the column “Specific terms”. ^b^ Terms as their appear in the GSEA’s output. ^c^ GSEA’s abbreviations are CGS = curated gene sets; GO = gene ontology; CP = canonical pathways. C2 through C5 refer to the MSigDB v7.3 collections (https://www.gsea-msigdb.org/gsea/msigdb/, accessed on 31 August 2023).

## Data Availability

Further research data can be provided upon request to the corresponding author.

## Supplementary Materials

The following supporting information can be downloaded at: https://www.mdpi.com/article/10.3390/ijms25010513/s1.
