# Supplementary figures and images for: Transcriptomic Analysis of the Aged Nulliparous Mouse Ovary Suggests a Stress State That Promotes Pro-Inflammatory Lipid Signaling and Epithelial Cell Enrichment
Source: Int J Mol Sci. 2023 Dec 30;25(1):513. doi: 10.3390/ijms25010513 (PMC10779227; doi:10.3390/ijms25010513)

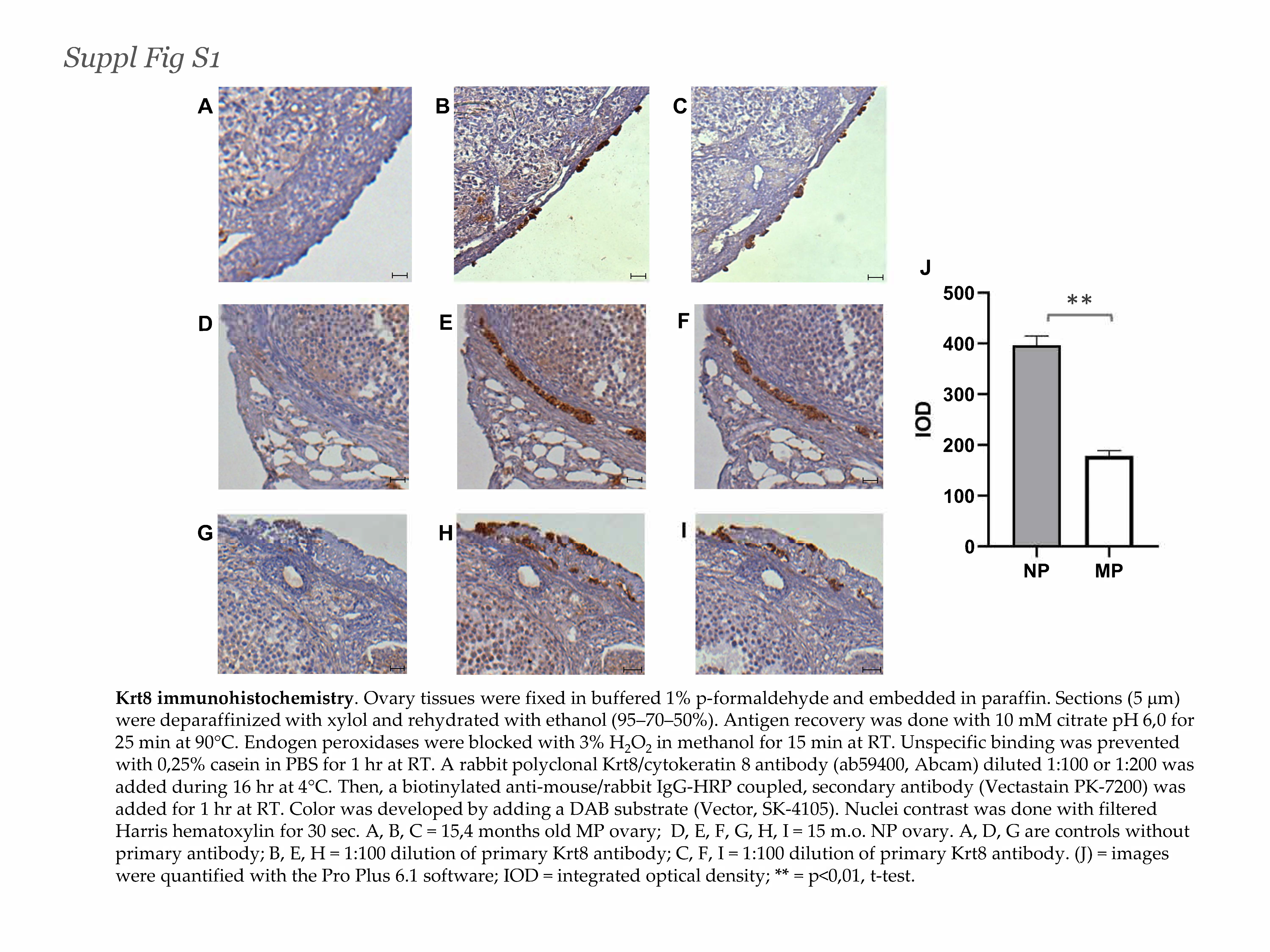

Supplement: Supplementary file 1 [file ijms-25-00513-s001.zip › Figure S1.png]

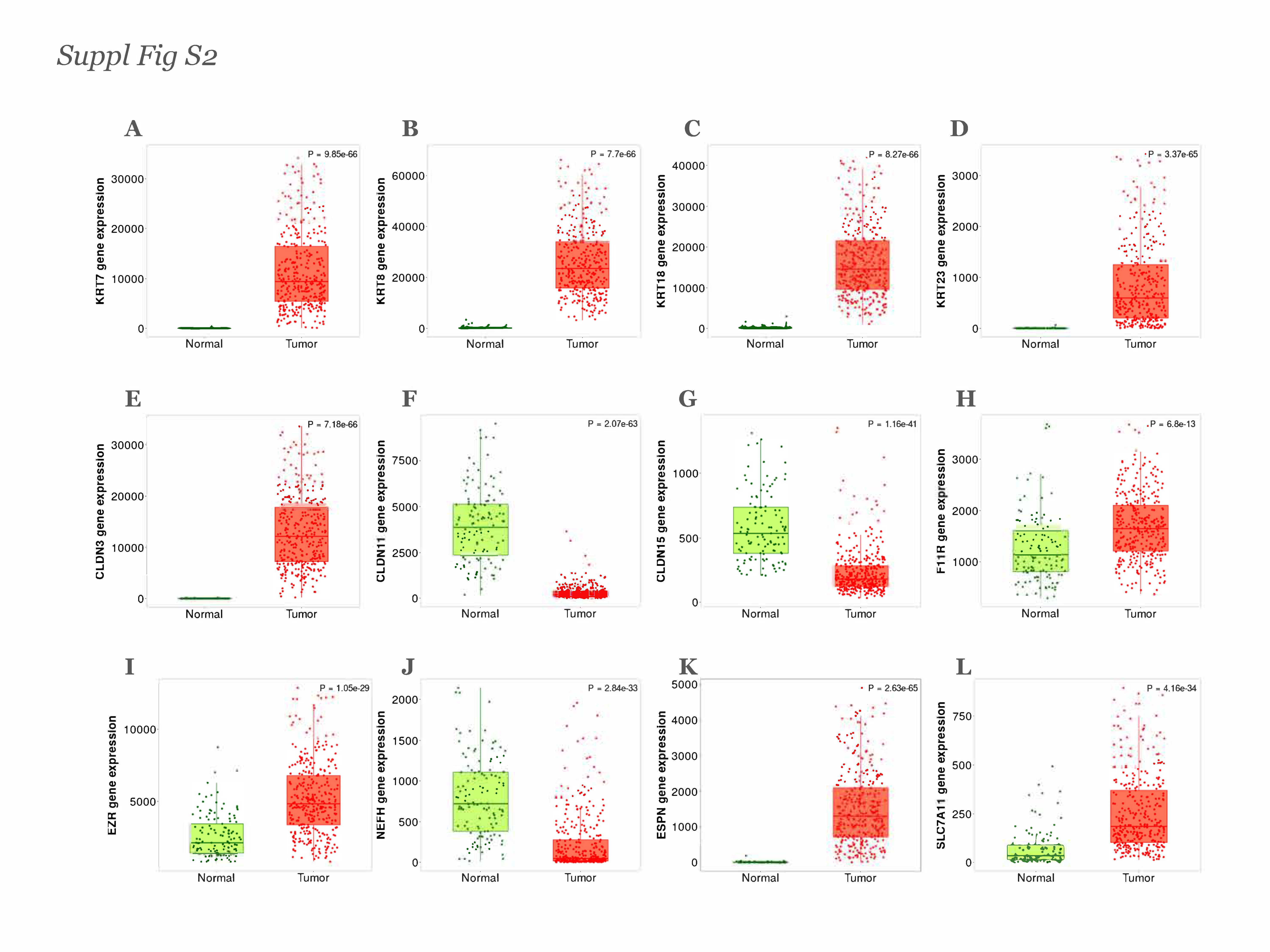

Supplement: Supplementary file 1 [file ijms-25-00513-s001.zip › Figure S2.png]
